# Predicting dementia using socio-demographic characteristics and the Free and Cued Selective Reminding Test in the general population

**DOI:** 10.1186/s13195-016-0230-x

**Published:** 2017-03-23

**Authors:** Thibault Mura, Marieta Baramova, Audrey Gabelle, Sylvaine Artero, Jean-François Dartigues, Hélène Amieva, Claudine Berr

**Affiliations:** 1 Neuropsychiatry: Epidemiological and Clinical Research, INSERM U1061, 34093 Montpellier, Cedex 5 France; 2 University of Montpellier, Montpellier, France; 3 Medical Information Department, University Hospital of Montpellier, Montpellier, France; 4 Memory Consultation, Centre Mémoire de Ressource et de Recherche, Gui de Chauliac University Hospital, Montpellier, France; 5 Memory Consultation, Centre Mémoire de Ressource et de Recherche, University Hospital, Bordeaux, France; 6 ISPED, Centre INSERM U1219, Bordeaux Population Health Research Center, Bordeaux, France; 7 University of Bordeaux,, Centre INSERM U1219, Bordeaux Population Health Research Center, Bordeaux, France

**Keywords:** Dementia, Prediction, Alzheimer disease, prodromal Alzheimer disease, preclinical Alzheimer disease, Early clinical trial

## Abstract

**Background:**

Our study aimed to determine whether the consideration of socio-demographic features improves the prediction of Alzheimer’s dementia (AD) at 5 years when using the Free and Cued Selective Reminding Test (FCSRT) in the general older population.

**Methods:**

Our analyses focused on 2558 subjects from the prospective Three-City Study, a cohort of community-dwelling individuals aged 65 years and over, with FCSRT scores. Four “residual scores” and “risk scores” were built that included the FCSRT scores and socio-demographic variables. The predictive performance of crude, residual and risk scores was analyzed by comparing the areas under the ROC curve (AUC).

**Results:**

In total, 1750 subjects were seen 5 years after completing the FCSRT. AD was diagnosed in 116 of them. Compared with the crude free-recall score, the predictive performances of the residual score and of the risk score were not significantly improved (AUC: 0.83 vs 0.82 and 0.88 vs 0.89 respectively).

**Conclusion:**

Using socio-demographic features in addition to the FCSRT does not improve its predictive performance for dementia or AD.

**Electronic supplementary material:**

The online version of this article (doi:10.1186/s13195-016-0230-x) contains supplementary material, which is available to authorized users.

## Background

The early identification of preclinical forms of Alzheimer’s disease (AD) has been the focus of much research over the last two decades. Indeed, because the AD pathophysiological process begins several years or decades before the clinical diagnosis [1–3], it seems more promising, for treating the disease, to act upstream of the clinical stage, before the installation of irreversible damage [4]. The development of such therapies requires the early identification of patients with AD at the predementia stage. In accordance with these conceptual advances, AD diagnosis has been redefined recently in the context of research. It now requires, for its typical form, the combination of amnesic syndrome of hippocampal type and a pathophysiological AD biomarker, such as cerebrospinal fluid proteins (decreased Aβ42 and increased T-tau and P-tau) or amyloid plaques detected by PET imaging with a specific tracer [5]. However, the measurement of such biomarkers cannot be generalized to the entire population because of the potential side effects of the used methods (exposure to radiation for brain imaging; pain and risks related to invasive procedures for cerebrospinal fluid collection) and because of their ever-increasing cost [6]. A possible alternative could be a large screening using a noninvasive and cheap tool [7], such as a neuropsychological test. The Free and Cued Selective Reminding Test (FCSRT) [8], which has been recommended for assessing amnesic syndrome of hippocampal type [5, 9], could be potentially useful in this context. Indeed, in a first study conducted in general population [10], the FCSRT exhibited good sensitivity and specificity for AD prediction at 5 years (92% and 64% respectively for the FCSRT free recall), but showed a poor positive predictive value of about 8%.

To our knowledge, no study has tried to improve the predictive performances of a neuropsychological test by combining the test results with some readily available information, such as socio-demographic data. Socio-demographic features (sex, age and education) strongly influence cognitive scores [11–16] and the dementia risk [17]. Combining these characteristics with the results of an episodic memory test could therefore improve dementia prediction. Two types of algorithm can be used for this prediction. The predictive information contained in socio-demographic variables can be added to the neuropsychological test score to generate a predictive “risk score”, built according to the recommendations of the international literature [18]. Alternatively, an algorithm can be developed to allow the interpretation of the neuropsychological test scores as a function of the socio-demographic characteristics. A “residual score” can thus be calculated that corresponds to the difference between the observed and the expected scores for a subject of a given sex, age and education level. Such a “residual score” was recently proposed by Reed et al. [19] for quantifying the cognitive reserve [20] from the scores of an episodic memory test after removing the variability due to socio-demographic factors and level of brain pathology.

The objective of this study was therefore to determine whether the addition of socio-demographic factors to the FCSRT score to generate a risk score or the use of a residual score could improve the prediction of dementia and/or AD at 3 and 5 years compared with the FCSRT crude scores alone, in a population-based cohort of older subjects.

## Methods

### Population and study design

**Fig. 1:**
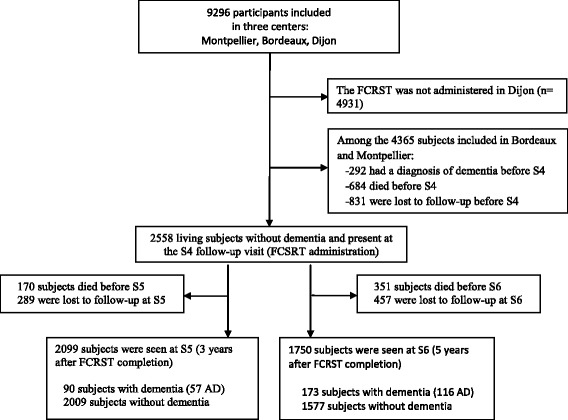
Study flow chart. *AD* Alzheimer’s disease, *FCSRT* Free and Cued Selective Reminding Test, *S4* visit with FCRST administration, *S5* 3 years after FCRST administration, *S6* 5 years after FCRST administration

Data were extracted from the multi-site prospective Three-City Study (3C) cohort study on 9294 community-dwelling persons aged 65 years and over recruited from the electoral rolls of three French cities between 1999 and 2001 [21]. The 3C protocol was approved by the Ethical Committee of the University Hospital of Bicêtre (France) and written informed consent was obtained from each participant. Socio-demographic characteristics, health status and lifestyle information were collected using standardized questionnaires during face-to-face interviews. Subjects were seen again at 2 years (S1), 4 years (S2), 5 years (S3), 7 years (S4), 10 years (S5) and 12 years (S6) after inclusion. Because the FCSRT was administered only to participants from the Montpellier and Bordeaux 3C centers at the S4 visit, the current study used data for 2558 subjects from these two cities who were nondemented and completed the cognitive tests at S4 (baseline of our analyses). Among these subjects, 459 were excluded (170 dead and 289 lost to follow-up between S4 and S5) for the prediction of dementia at 3 years after FCSRT completion, and 808 (351 dead and 457 lost to follow-up between S4 and S6) for the prediction of dementia at 5 years after the FCSRT (Fig. 1).

### FCSRT administration and scores

The French version [22] of the FCSRT [8, 22] was administered at S4 for the first time in the Montpellier center and for the second time in the Bordeaux center. The test assesses verbal episodic memory. The neuropsychological test begins with an encoding phase during which the patient has to learn 16 words, four at a time (presented on a card). Each word belongs to a different semantic category. The subject is asked to say the name of the item corresponding to a specific semantic category (e.g., “what is the name of this fruit?”). After all four items are identified correctly, the card is removed and their immediate cued recall is tested by presenting the cues again in order to control for encoding. Once the immediate recall of a group of four items is completed, the next set of four items is presented. After the encoding phase is completed, the participant is asked to recall as many words as possible in 2 minutes (free recall). The neuropsychologist then provides a cue (word category) for each nonretrieved word to help the patient recalling the remaining words. Free and cued recalls are repeated three times. The delayed recall phase is performed 20 minutes later, also with a free and a cued recall.

For our analyses, we used the “free recall score” (sum of the number of words retrieved at the three free recall trials), the “total recall score” (sum of the three free + cued recall trials) (both scores range from 0 to 48), the “delayed free recall score” (number of words retrieved at the delayed free recall trial) and the “delayed total recall score” (free + cued delayed recall trial) (both scores range from 0 to 16).

### Diagnosis of dementia

Dementia was diagnosed in two steps at each follow-up visit. Subjects at risk were identified by the 3C neuropsychologist on the basis of the results of the Mini-Mental State Examination (MMSE) [23] and the Isaacs set test (IST) [24]. The IST, consisting of generating words belonging to four semantic categories (cities, fruits, animals and colors) in 15 seconds, measured semantic verbal fluency ability. These participants were then examined by a 3C physician who confirmed or not the diagnosis and severity of the disease. The physician was blind to the FCSRT scores. Finally, all diagnoses were reviewed by a panel of five neurologists, independent of the 3C investigators. The panel examined all available information, including the FCSRT scores, and agreed on the presence or absence of dementia, according to the DSM-IV TR criteria [25], and on its etiology using the National Institute of Neurological and Communicative Disorders and Stroke–Alzheimer's Disease and Related Disorders Association criteria [26] for probable and possible AD. When we further analysed the prediction of AD, subjects with a diagnosis of non-AD dementia were not excluded but were included in a group which contained both patients without dementia and patients with non-AD dementia.

### Statistical analysis

The participants’ characteristics were described using frequency and proportion for categorical variables and mean and standard deviation (SD) for continuous variables. Comparisons between groups were performed using the Student’s *t* test for the FCSRT scores and the chi-square test for percentages of missing data (subjects who did not begin or complete the FCSRT at S4).

To construct the FCSRT “residual scores”, the crude scores were modeled using linear regression models that included, as independent variables, age, age-squared, sex and education level (primary school: 0–5 years of education; vocational school certificate; French junior-school diploma; French high-school diploma; graduate studies). The total proportion of variance of the crude scores explained by the linear model was expressed using the coefficient of determination, denoted *R* ^2^. The estimated beta coefficients were used to generate the estimated FCSRT scores for each individual. The “residual scores” were then defined and computed as the difference between the estimated and the crude FCSRT scores. Consequently, the variability of these *“*residual scores” corresponds to the FCSRT residual variance after removing the part of variance associated with the socio-demographics variables [19].

The dementia and AD “risk scores” were constructed using a logistic regression model. The probability of dementia or AD at 3 and 5 years (S5 and S6 visits) after FCSRT completion, and between these two visits, was modeled as a function of the FCSRT crude scores, age, sex, education level and interaction between the FCSRT crude scores and the three socio-demographic characteristics (age, sex and education level). Regression coefficients were used to compute a risk score for each subject. To avoid over-fitting, the risk scores were evaluated using cross-validation methods by splitting the sample into two, according to the inclusion center [27]. Thus, the risk score coefficients were estimated using only the Bordeaux data (training dataset) because of the larger number of patients with dementia in this center. The risk score predictive performances were evaluated using the Montpellier data (testing dataset). A sensibility analysis was conducted by inverting the cities for the training and testing dataset*.*

The performance of the “crude scores”, “residual scores” and “risk scores” for the prediction of dementia and AD at 3 and 5 years, and between 3 and 5 years, was assessed using receiver operating curve (ROC) analysis [28]. The area under the curve (AUC) values were compared using the nonparametric method described by DeLong et al. [29].

Analyses were performed with a bilateral alpha level of 0.05 using SAS software, version 9.1 (SAS Institute, Cary, NC, USA).

## Results

At the S4 follow-up visit (baseline of our analyses), the mean age of the 2558 participants was 80 years (SD: 4.7), 63.8% were women, 27.3% had 0–5 years of education, 17.0% had a French junior-school diploma (9 years of education), 11.1% had a vocational school certificate (10–11 years of education), 14.9% had a French high-school diploma (12 years of education) and 29.7% had a graduate diploma (at least 14 years of education).

At S5 (3 years after FCSRT completion) 90 participants had developed dementia (AD *n* = 57), and at S7 (5 years after FCSRT completion) the cumulative number of incident cases of dementia (over the 3 and 5 years) was 173 (AD *n* = 116).

**Fig. 2:**
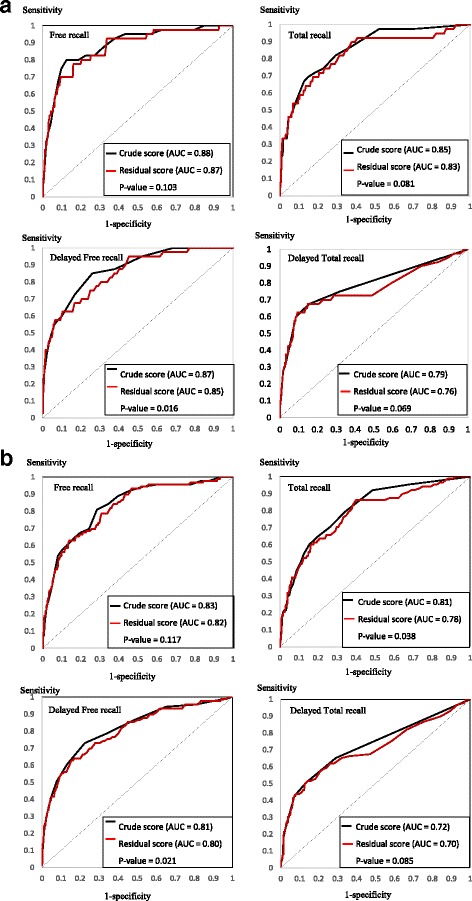
ROC curves representing the FCSRT crude and residual scores for the prediction of Alzheimer's disease at **a** 3 years and **b** 5 years *AUC* area under the curve, *FCSRT* Free and Cued Selective Reminding Test

The coefficients of the linear regressions used to produce the expected FCSRT scores according to age, age-squared, sex and level of education are shown in Additional file 1: Table S1. These models explained from *R* ^2^ = 4.5% to *R* ^2^ = 10.6% of the total variance of the different FCSRT scores. The “crude scores”, the “residual scores” (differences between crude and estimated FCSRT scores) and the percentage of “refusal or abandon” (subjects who did not begin or complete the neuropsychological test at S4) relative to the occurrence of dementia and AD at 3 and 5 years are presented in Table 1. Comparisons of the AUC for the ROC curves of the crude and residual scores (Table 2 and Fig. 2a, b) showed that, compared with the crude scores, the residual scores did not improve the prediction of dementia or AD occurrence at 3 or 5 years, and between 3 and 5 years, whichever FCSRT score was considered.

**Table 1:** Residual and crude FCSRT scores according to the occurrence of dementia and Alzheimer's disease at 3 years (S5) and 5 years (S6) after test completion (all comparison *p* < 0.01^a^)

	Dementia at 3 years		Dementia between 3 and 5 years		Dementia at 5 years		AD at 3 years		AD between 3 and 5 years		AD at 5 years	
	No (*N* = 2009)	Yes (*N* = 90)	No (*N* = 1577)	Yes (*N* = 83)	No (*N* = 1577)	Yes (*N* = 173)	No (*N* = 2042)	Yes (*N* = 57)	No (*N* = 1601)	Yes (*N* = 59)	No (*N* = 1634)	Yes (*N* = 116)
FCSRT free recall												
Score mean (SD)	25.2 (6.3)	16.2 (6.0)	25.9 (6.1)	18.2 (6.4)	25.9 (6.1)	17.2 (6.2)	25.1 (6.4)	14.5 (5.9)	25.7 (6.2)	19.1 (6.2)	25.7 (6.2)	17.0 (6.5)
Residual score mean (SD)	–0.6 (6.0)	–8.7 (6.7)	0.0 (5.7)	–7.1 (6.3)	0.0 (5.7)	–7.9 (6.5)	–0.7 (6.0)	–10.5 (6.3)	–0.2 (5.9)	–6.4 (6.1)	–0.2 (5.9)	–8.3 (6.5)
Missing data^b^, *n* (%)	143 (7.1%)	24 (26.7%)	88 (5.6%)	16 (19.2%)	88 (5.6%)	40 (23.1%)	149 (7.3%)	17 (29.8%)	94 (5.8%)	10 (16.9%)	101 (6.2%)	27 (23.3%)
FCSRT total recall												
Score mean (SD)	44.9 (4.2)	38.3 (7.7)	45.3 (3.9)	41.7 (5.2)	45.3 (3.9)	40.0 (6.7)	44.9 (4.2)	36.4 (7.7)	45.2 (4.0)	41.3 (5.4)	45.2 (4.0)	39.1 (6.9)
Residual score mean (SD)	–0.3 (4.0)	–6.4 (7.9)	0.0 (3.7)	–3.2 (5.1)	0.0 (3.7)	–4.8 (6.8)	–0.3 (4.1)	–8.3 (7.8)	–0.1 (3.9)	–3.7 (5.2)	–0.1 (3.9)	–5.8 (6.8)
Missing data^b^, *n* (%)	153 (7.6%)	25 (27.8%)	95 (6.0%)	16 (19.2%)	95 (6.0%)	41 (23.7%)	159 (7.8%)	18 (31.6%)	101 (6.3%)	10 (16.9%)	116 (7.1%)	28 (24.1%)
FCSRT delayed free recall												
Score mean (SD)	10.1 (2.7)	6.1 (3.6)	10.41 (2.5)	7.2 (3.3)	10.41 (2.5)	6.7 (3.5)	10.1 (2.7)	5.0 (3.4)	10.3 (2.6)	7.5 (3.3)	10.3 (2.6)	6.4 (3.6)
Residual score mean (SD)	–0.3 (2.6)	–3.9 (3.8)	0.0 (2.4)	–3.0 (3.2)	0.0 (2.4)	–3.4 (3.5)	–0.3 (2.6)	–5.0 (3.6)	–0.1 (2.5)	–2.7 (3.2)	–0.1 (2.5)	–3.8 (3.6)
Missing data^b^, *n* (%)	148 (6.8%)	24 (26.7%)	91 (5.3%)	16 (19.2%)	91 (5.3%)	40 (23.1%)	145 (7.1%)	17 (29.8%)	89 (5.6%)	10 (16.9%)	96 (5.9%)	27 (23.3%)
FCSRT delayed total recall												
Score mean (SD)	15.3 (1.4)	13.1 (3.1)	15.4 (1.3)	14.4 (2.0)	15.4 (1.3)	13.8 (2.7)	15.3 (1.4)	12.4 (3.4)	15.4 (1.4)	14.3 (2.1)	15.4 (1.4)	13.4 (2.9)
Residual score mean (SD)	–0.1 (1.4)	–2.1 (3.2)	0.0 (1.3)	–0.9 (2.0)	0.0 (1.3)	–1.5 (2.7)	–0.1 (1.4)	–2.9 (3.5)	–0.0 (1.3)	–1.1 (2.1)	–0.0 (1.3)	–1.9 (2.9)
Missing data^b^, *n* (%)	137 (6.8%)	24 (26.7%)	83 (5.3%)	16 (19.2%)	83 (5.3%)	40 (23.1%)	155 (7.6%)	17 (29.8%)	97 (6.1%)	10 (16.9%)	105 (6.4%)	27 (23.3%)

^a^ For the comparison between demented vs nondemented or AD vs non-AD subjects: all *p* ≤ 0.01

^b^ Subjects alive and present at the S4 (baseline of our analyses) visit who did not complete the neuropsychological test (refusal or abandon)

*FCSRT* Free and Cued Selective Reminding Test, *SD* standard deviation

**Table 2:** AUC comparisons between the residual and crude FCSRT scores for the prediction of dementia and Alzheimer's disease at 3 years (S5) and 5 years (S6) after test completion

	Dementia at 3 years		Dementia between 3 and 5 years		Dementia at 5 years		AD at 3 years		AD between 3 and 5 years		AD at 5 years	
	AUC (CI)	*p* value	AUC (CI)	*p* value	AUC (CI)	*p* value	AUC (CI)	*p* value	AUC (CI)	*p* value	AUC (CI)	*p* value
FCSRT free recall												
Crude score	0.85 (0.79–0.90)	0.010	0.81 (0.75–0.87)	0.253	0.83 (0.79–0.88)	0.004	0.88 (0.81–0.95)	0.103	0.78 (0.70–0.86)	0.414	0.83 (0.78–0.88)	0.117
Residual score	0.82 (0.75–0.88)		0.80 (0.74–0.87)		0.81 (0.77–0.86)		0.87 (0.80–0.94)		0.78 (0.68–0.85)		0.82 (0.77–0.88)	
FCSRT total recall												
Crude score	0.79 (0.73–0.86)	0.001	0.75(0.68–0.82)	0.030	0.78 (0.73–0.82)	0.001	0.85 (0.78–0.93)	0.081	0.76 (0.68–0.84)	0.181	0.81 (0.75–0.86)	0.038
Residual score	0.75 (0.68–0.82)		0.72(0.65–0.79)		0.74 (0.69–0.79)		0.83 (0.75–0.91)		0.74 (0.65–0.82)		0.78 (0.73–0.84)	
FCSRT delayed free recall												
Crude score	0.81 (0.74–0.87)	0.001	0.78(0.71–0.85)	0.103	0.80 (0.75–0.85)	0.001	0.87 (0.80–0.94)	0.016	0.76 (0.68–0.84)	0.286	0.81 (0.76–0.87)	0.021
Residual score	0.77 (0.71–0.84)		0.77 (0.70–0.83)		0.78 (0.73–0.82)		0.85 (0.77–0.93)		0.75 (0.66–0.82)		0.80 (0.74–0.86)	
FCSRT delayed total recall												
Crude score	0.72 (0.65–0.79)	0.001	0.67(0.60–0.74)	0.261	0.70 (0.65–0.75)	0.001	0.79 (0.70–0.87)	0.069	0.66 (0.58–0.75)	0.376	0.72 (0.66–0.78)	0.085
Residual score	0.67 (0.60–0.75)		0.65 (0.58–0.72)		0.66 (0.61–0.71)		0.76 (0.67–0.85)		0.64 (0.56–0.73)		0.70 (0.64–0.76)	

*AUC* area under the curve, *CI* confidence interval. *FCSRT* Free and Cued Selective Reminding Test

The construction of the “risk scores” was based on the beta coefficients from logistic regression analyses performed using the Bordeaux center data (training dataset). Each risk score included a given FCSRT score, age, sex, education level and the interaction between these three variables and the FCSRT score. Coefficients of logistic regression used for the construction of risk scores are shown in Additional file 2: Table S2. The performances of the risk scores for the prediction of dementia or AD were evaluated using the Montpellier center data (testing dataset) (as recommended in [27]). Comparison of the AUC for the risk scores vs crude scores showed that the risk scores did not improve the prediction of dementia and AD at 3 and 5 years, and between 3 and 5 years (Table 3). The sensibility analysis conducted by inverting the cities for the training and testing dataset led to the same conclusion (data not shown).

**Table 3:** AUC comparisons between the cross-validated risk scores^a^ and the FCSRT crude scores for the prediction of dementia and Alzheimer's disease at 3 and 5 years in the Montpellier center (testing dataset)

	Dementia at 3 years		Dementia between 3 and 5 years		Dementia at 5 years		AD at 3 years		AD between 3 and 5 years		AD at 5 years	
	AUC (CI)	*p* value	AUC (CI)	*p* value	AUC (CI)	*p* value	AUC (CI)	*p* value	AUC (CI)	*p* value	AUC (CI)	*p* value
FCSRT free recall												
Crude score	0.89 (0.80–0.99)	0.44	0.87 (0.79–0.95)	0.80	0.89 (0.83–0.95)	0.84	0.92 (0.82–1.00)	0.79	0.85 (0.74–0.95)	0.92	0.88 (0.81–0.96)	0.34
Risk score	0.89 (0.79–0.98)		0.82 (0.73–0.91)		0.89 (0.83–0.95)		0.92 (0.81–1.00)		0.84 (0.74–0.95)		0.89 (0.82–0.97)	
FCSRT total recall												
Crude score	0.89 (0.79–0.98)	0.11	0.80 (0.70–0.89)	0.93	0.84 (0.77–0.91)	0.82	0.91 (0.80–1.00)	0.18	0.79 (0.67–0.91)	0.42	0.84 (0.75–0.93)	0.95
Risk score	0.86 (0.75–0.96)		0.76 (0.66–0.86)		0.83 (0.76–0.90)		0.88 (0.76–1.00)		0.76 (0.64–0.88)		0.84 (0.75–0.93)	
FCSRT delayed free recall												
Crude score	0.86 (0.76–0.97)	0.91	0.81 (0.71–0.90)	0.53	0.83 (0.77–0.90)	0.27	0.92 (0.82–1.00)	0.94	0.78 (0.66–0.90)	0.66	0.84 (0.76–0.93)	0.04
Risk score	0.86 (0.76–0.96)		0.75 (0.65–0.85)		0.85 (0.78–0.91)		0.92 (0.82–1.00)		0.77 (0.65–0.90)		0.86 (0.78–0.94)	
FCSRT delayed total recall												
Crude score	0.86 (0.75–0.96)	0.11	0.71 (0.60–0.82)	0.33	0.77 (0.69–0.85)	0.64	0.88 (0.75–1.00)	0.00	0.69 (0.56–0.82)	0.22	0.77 (0.67–0.86)	0.35
Risk score	0.72 (0.59–0.85)		0.73 (0.63–0.83)		0.79 (0.71–0.86)		0.69 (0.53–0.85)		0.75 (0.63–0.88)		0.81 (0.72–0.90)	

^a^The cross-validated risk scores were constructed using the Bordeaux data (training dataset)

*AUC* area under the curve, *CI* confidence interval. *FCSRT* Free and Cued Selective Reminding Test

## Discussion

Here, we show that the use of “risk scores” or “residual scores” that take into account sex, age and education level does not improve the FCSRT performance for the prediction of dementia and AD at 3 and 5 years compared with the crude FCSRT scores. Therefore, the FCSRT on its own, without the addition of socio-demographic data, is sufficient for the prediction of dementia and AD in the general population.

Our study has several strengths. First, we conducted this analysis on data from the 3C trial, a large cohort of older subjects recruited from the general population in France. This enabled us to have sufficient numbers of patients who developed dementia or AD during the follow-up for a prediction study that included a cross-validation of the risk scores. Second, we used the FCSRT [8]. This neuropsychological test is recommended for assessing amnesic syndrome of hippocampal type [5] and has been frequently reported to be efficient also in the context of AD detection and prediction [9, 30–32]. In agreement, our study confirmed the previous results on FCSRT prediction of dementia in the Bordeaux cohort of the 3C [10] and showed the better performance of the free recall score for the prediction of dementia and AD, as reported also by the GuidAge study in patients with memory complaints [30]. We used in this study the validated French version of the FCRST [22] which has been used in most French studies analyzing the FCRST (PREAL study [9], Three-City Study [10] or CONSTANCES study [16]). This version differs from the original version in the presentation, during the encoding phase of the test, with cards displaying words (4 × 4) adapted to the French-speaking population rather than cards displaying pictures. In two French studies [9, 10] which have determined a cutoff value to predict dementia, these values were lower than the one proposed by Grobber et al. [33]. More precisely, Sarazin et al. [9] proposed a free recall score ≤ 17 for the prediction of AD at 3 years in MCI subjects, whereas Grobber et al. [33] proposed a score ≤ 24 to identify very mild dementia in primary care. Furthermore, Auriacombe et al. [10] proposed a free recall score ≤ 16 for the prediction of dementia at 2 years, and a score ≤ 24 for the prediction at 5 years in the general population. This means that the French version of the test is calibrated on a more difficult basis than the original version, and explains why subjects who became demented during our study appear to have an initial FCSRT score similar to the scores of already demented patients in the publication by Grober et al. [33].

A point which could be raised concerning the prediction performances of the “residual scores” is the part of the variance explained by socio-demographics factors. This part varies from 10.6% for the free recall score to 4.5% for the delayed total recall score. These percentages are consistent with figures published in the general population [16]. The smaller amount of explained variance for the total recall and delayed total recall scores can be explained by the ceiling effect that affects these two scores. Indeed, if a large number of subjects have the maximum score, the part of variance that might be explained by socio-demographic factors is automatically reduced. Conversely, the free recall and delayed free recall scores do not exhibit a ceiling effect. Another explanation could be the relative homogeneity of our sample (all subjects were older than 70 years) that did not allow fully capturing the cognitive variability between younger and older subjects.

In our study, 7–8% of participants did not complete the FCSRT test. This should not be considered a limitation of the study. Indeed, the interruption or refusal during the execution of a test is a reality that cannot be avoided in clinical practice. Moreover, our data indicate that almost one-third of noncompleters developed AD in the next 3 years (Table 1). This behavior could therefore be considered a risk factor for AD. This result is consistent with a previous publication showing that refusing neuropsychological tests is associated with poorer cognitive functioning [34].

For this study, we did not have access to postmortem data to confirm the diagnosis of AD or dementia. Nevertheless, all diagnoses were reviewed by a panel of independent experts who had access to all data, including the FCSRT scores. Consequently, the clinical diagnoses and FCSRT results were not independent, which could have overestimated FCSRT predictive performance. However, it is unlikely that this potential classification bias influenced the comparison of the crude, residual and risk scores. Moreover, for AD prediction, we grouped subjects with other dementia types in the non-AD group, together with nondemented subjects. This choice might have decreased FCSRT predictive performance, but should not have influenced the comparison between scores.

Concerning the risk scores, their construction and assessment using the same dataset generally leads to overfitting the model and overestimating their performances. Therefore, we split our data into two datasets, based on the inclusion center (Bordeaux and Montpellier), for their construction and cross-validation, as recommended by Altman et al. [27]. This analysis showed that the risk scores did not predict dementia or AD better than the crude scores.

The absence of improvement of the test predictive performances when the socio-demographic variables are taken into account could also bring new insight into the question of the cognitive reserve. Reed et al. [19] suggested including socio-demographic factors in a residual score for AD prediction because they thought that the neuropsychological test scores should be corrected for age, sex and education to better measure the cognitive reserve. Conversely, our results suggest that this is not the case. Reed et al.’s [19] approach was based on the hypothesis that the cognitive reserve could be better estimated by removing the parts of variability of a neuropsychological test due to socio-demographic features and brain damage load. We did not have information about the severity of brain damage in our population, a major issue in their hypothesis [19]. Nonetheless, our results stress that this hypothesis needs to be further investigated before being accepted.

Another new finding of our study is that FCSRT crude scores exhibit good performance for the prediction of dementia between 3 and 5 years independently of the cases diagnosed in the first 3 years. Although it may seem difficult in practice to differentiate subjects who will convert before and after 3 years, these results explore the maintenance of performance of the neuropsychological test several years after the examination. It will be interesting in future research to study the prediction of dementia and/or AD even earlier, 5 to 10 years before the diagnosis of dementia. In this context our results will not necessarily apply to this issue and will have to be re-evaluated

A practical implication of our results is that, in the context of a therapeutic trial concerning the early stages of dementia/AD (i.e., before the clinical signs/diagnosis), the FCSRT could be used alone, without any information about socio-demographic features, to identify the target population at very high risk of dementia and/or AD. It could be used with a single threshold (e.g., a free recall score ≤ 22, as proposed by Auriacombe et al. [10]), without norms by sex, age and education, despite the strong FCSRT dependence on these features. In the future, other methods, such as the simultaneous use of several cognitive scores or the analysis of FCSRT longitudinal variations during a repeated follow-up, could be explored to improve FCSRT predictive performances.

## Conclusions

Our study shows that the FCSRT predicts dementia and AD independently from socio-demographic characteristics, and that the use of this information does not improve prediction of dementia when using FCSRT results in the general population.

## Additional files

Additional file 1: is **Table S1.** presenting coefficients for the linear regression analyzing the relation between FCSRT scores and age, age-squared, sex and education. (DOCX 20 kb)

Additional file 2: is **Table S2.** presenting coefficients of logistic regression used for the construction of risk scores of Alzheimer’s dementia at 3 years and at 5 years based on age, sex, education, FCSRT scores and the interaction between socio-demographics factors and FCSRT scores. (DOCX 29 kb)

## Abbreviations

AD: Alzheimer’s disease
AUC: Area under the curve
FCSRT: Free and Cued Selective Reminding Test
MMSE: Mini Mental State Examination
*R*^2^: Coefficient of determination
ROC: Receiver operating curve
SD: Standard deviation
