# Supplementary material for: Predicting dementia using socio-demographic characteristics and the Free and Cued Selective Reminding Test in the general population
Source: Alzheimers Res Ther. 2017 Mar 23;9:21. doi: 10.1186/s13195-016-0230-x (PMC5364571; doi:10.1186/s13195-016-0230-x)
Supplement: Supplementary file 1 — is Table S1. presenting coefficients for the linear regression analyzing the relation between FCSRT scores and age, age-squared, sex and education. (DOCX 20 kb) [file 13195_2016_230_MOESM1_ESM.docx]

Additional Table 1.

Coefficients of the linear regression analysis to model the interaction between FCSRT scores and age, age squared, sex and education.

|  | **FCSRT free recall** | **FCSRT total recall** | **FCSRT delayed free recall** | **FCSRT delayed total recall** |
| --- | --- | --- | --- | --- |
|  | Coefficient (SE) | Coefficient (SE) | Coefficient (SE) | Coefficient (SE) |
| Intercept | 31.27 (44.7) | 23.86 (29.2) | 16.45 (18.9) | 15.41 (10.0) |
| Age | 0.03 (1.1) | 0.60 (0.7) | -0.06 (0.5) | 0.02 (0.2) |
| Age squared | -0.00 (0.0) | -0.00 (0.0) | -0.00 (0.0) | -0.00 (0.0) |
| Sex, Women | 2.53^*^  (0.3) | 0.99^*^ (0.2) | 0.80^*^ (0.1) | 0.30^*^  (0.1) |
| Education |  |  |  |  |
| 0 to 5 yrs of education | ref | ref | ref | ref |
| Vocational school certificate (11 yrs) | 1.12^b^ (0.5) | 1.26^*^  (0.4) | 0.39 (0.2) | 0.31^*^ (0.1) |
| French junior-school diploma (9 yrs) | 1.58^a^ (0.5) | 1.54^*^  (0.3) | 0.59^*^  (0.2) | 0.37^*^ (0.1) |
| French high-school diploma (12 yrs) | 2.66^a^  (0.5) | 1.81^*^ (0.3) | 0.87^*^ (0.2) | 0.39^*^ (0.1) |
| Graduate studies (≥14 yrs) | 3.45^a^ (0.4) | 2.27 ^*^  (0.3) | 1.16^*^ (0.2) | 0.57 (0.1) |
| R², % | 10.6 | 7.5 | 8.1 | 4.5 |

^*^ Coefficient significantly different from 0 (p value ≤0.05)

Ref, reference; SE, standard error; yrs, years.; R^2^ : Coefficient of determination
