# Supplementary material for: Predicting dementia using socio-demographic characteristics and the Free and Cued Selective Reminding Test in the general population
Source: Alzheimers Res Ther. 2017 Mar 23;9:21. doi: 10.1186/s13195-016-0230-x (PMC5364571; doi:10.1186/s13195-016-0230-x)
Supplement: Supplementary file 2 — is Table S2. presenting coefficients of logistic regression used for the construction of risk scores of Alzheimer’s dementia at 3 years and at 5 years based on age, sex, education, FCSRT scores and the interaction between socio-demographics factors and FCSRT scores. (DOCX 29 kb) [file 13195_2016_230_MOESM2_ESM.docx]

Additional Table 2.

Coefficients of logistic regression used for the construction of risk scores of Alzheimer Dementia at 3 years and at 5 years based on age, sex, education, FCSRT scores and the interaction between socio-demographics factors and FCSRT scores

|  | Alzheimer Dementia  **at 3 years** | | | |  | Alzheimer Dementia **at 5 years** | | | |
| --- | --- | --- | --- | --- | --- | --- | --- | --- | --- |
|  | **FCSRT**  **Free recall** | **FCSRT**  **total recall** | **FCSRT delayed**  **free recall** | **FCSRT delayed total recall** |  | **FCSRT**  **Free recall** | **FCSRT**  **total recall** | **FCSRT delayed**  **free recall** | **FCSRT delayed total recall** |
|  | Coefficient(SE) | Coefficient(SE) | Coefficient(SE) | Coefficient(SE) |  | Coefficient(SE) | Coefficient(SE) | Coefficient(SE) | Coefficient(SE) |
| Intercept | -0.32 (10.18) | 19.58 (14.09) | 2.64 (4.70) | 19.81 (16.07) |  | 3.23 (6.67) | 35.43 (11.76)^*^ | 0.86 (5.33) | 24.68 (11.78)^*^ |
| Age | 0.00 (0.13) | -0.19 (0.17) | -0.04 (0.06) | -0.22 (0.20) |  | -0.03 (0.08) | -0.38 (0.14)^*^ | -0.01 (0.07) | -0.27 (0.15) |
| Sex (women) | 1.77 (0.77)^*^ | 0.56 (1.17) | 1.18 (0.48)^*^ | 1.48 (1.02) |  | 1.19 (0.49)^*^ | -0.05 (0.90) | 0.73 (0.47) | 1.03 (0.84) |
| Education |  |  |  |  |  |  |  |  |  |
| 0 to 5 years of primary school |  |  |  |  |  |  |  |  |  |
| Vocational school certificate (11 yrs) | -0.06 (1.56) | 0.06 (2.68) | -0.51 (0.99) | -1.15 (3.01) |  | -1.47 (1.20) | -1.11 (2.42) | -1.76 (1.40) | -2.83 (2.90) |
| French junior-school diploma (9 yrs) | 0.46 (1.29) | 4.77 (2.11) ^*^ | -0.35 (0.70) | 1.52 (2.01) |  | 0.27 (0.90) | 1.93 (1.77) | -0.32 (0.82) | -0.47 (1.70) |
| French high-school diploma(12 yrs) | -0.47 (1.12) | -2.41 (1.47) | 0.00 (0.75) | 0.11 (1.72) |  | 1.02 (0.93) | 0.52 (1.68) | 1.66 (0.89) | 3.37 (1.76)^*^ |
| Graduate studies (≥14 yrs) | -0.29 (1.06) | -2.53 (1.45) | 1.04 (0.65) | -1.48 (1.41) |  | 0.10 (0.76) | -0.42 (1.37) | 0.62 (0.73) | 0.43 (1.43) |
| Crude score | -0.44 (0.60) | -0.78 (0.35) ^*^ | -1.76 (0.72)^*^ | -2.24 (1.14) |  | -0.50 (0.35) | -1.08 (0.28)^*^ | -1.05 (0.70) | -2.42 (0.81)^*^ |
| **Interaction between Crude Scores and** |  |  |  |  |  |  |  |  |  |
| Age | 0.00 (0.01) | 0.01 (0.00) | 0.02 (0.01)^*^ | 0.02 (0.01)^*^ |  | 0.00 (0.00) | 0.01 (0.00)^*^ | 0.01 (0.01) | 0.03 (0.01)^*^ |
| Sex | -0.08 (0.04) | 0.00 (0.03) | -0.10 (0.06) | -0.07 (0.07) |  | -0.04 (0.02) | 0.01 (0.02) | -0.04 (0.06) | -0.05 (0.06) |
| Vocational school certificate (11 yrs) | 0.01 (0.09) | 0.00 (0.06) | 0.12 (0.13) | 0.09 (0.21) |  | 0.06 (0.05) | 0.02 (0.06) | 0.19 (0.15) | 0.18 (0.19) |
| French junior-school diploma (9 yrs) | -0.01 (0.07) | -0.11 (0.05) ^*^ | 0.08 (0.10) | -0.09 (0.14) |  | 0.00 (0.04) | -0.04 (0.04) | 0.07 (0.10) | 0.05 (0.11) |
| French high-school diploma(12 yrs) | 0.03 (0.06) | 0.06 (0.04) | 0.06 (0.11) | 0.00 (0.12) |  | -0.04 (0.05) | -0.01 (0.04) | -0.18 (0.12) | -0.22 (0.12) |
| Graduate studies (≥ 14 yrs) | -0.01 (0.06) | 0.05 (0.04) | -0.32 (0.13)^*^ | 0.06 (0.10) |  | -0.01 (0.04) | 0.00 (0.03) | -0.11 (0.09) | -0.05 (0.10) |

* Coefficient significantly different from 0, p<0.05
